# MultiFAR: Multidimensional information fusion with attention-driven representation learning for student performance prediction

**DOI:** 10.1371/journal.pone.0333099

**Published:** 2025-10-24

**Authors:** Mohd Fazil, Bader M. Albahlal

**Affiliations:** College of Computer and Information Sciences, Imam Mohammad Ibn Saud Islamic University (IMSIU), Riyadh, Kingdom of Saudi Arabia; University of Queensland - Saint Lucia Campus: The University of Queensland, AUSTRALIA

## Abstract

The advancement in computing technology, online learning platforms, and pedagogical tools enable educators and learners to connect without temporal and geographical boundaries. The existing deep learning models to predict student performance are either simple recurrent neural networks or artificial neural networks employing demographic and hand-crafted features. This manuscript proposes a model, MultIFAR, that infuses multi-dimensional information representing different aspects of student behavior with an attention-driven deep learning model integrating bidirectional long short-term memory and convolutional networks to learn student representation efficiently. MultIFAR employs student demographic, assessment, and VLE-interaction to understand different aspects of student behavior from multifaceted data. MultIFAR includes bidirectional long short-term memory to process and capture patterns from demographic, assessment, and interaction information. The model applies a convolutional operation on the daily interaction information with the VLE. We also implement the attention mechanism to assign weight to significant features. The empirical evaluation over the Open University Learning Analytics (OULA) dataset establishes the efficacy of MultIFAR against the state-of-the-art approaches and baseline methods. Considering accuracy, MultIFAR reports results from 80.31% to 97.12% over the four different problems of student performance prediction. The ablation analysis reveals that diurnal interaction shows the highest, whereas demographic attributes show the least impact on MultIFAR accuracy. We also extend MultIFAR to predict at-risk and high-performing students early. We also evaluate the model over the balanced dataset and multiclass scenario.

## Introduction

In this century, rapid advancement in computing technology has influenced every aspect of human life. Likewise, the education sector is also influenced by the development of various tools and platforms such as Blackboard and Moodle. These tools change the student’s learning behavior. These tools have facilitated access to educational content without geographical and temporal limitations. Millions of students access educational content through the massive open online courses (MOOCs) and virtual learning environments. The students’ interactions with digital learning platforms generate a significant dataset that we can analyze to gain insights into learner behavior and identify the factors influencing student performance. The existing literature includes approaches where researchers have used VLE-generated data to comprehend the learning process and further presented statistical analysis, machine learning, and data mining techniques to automatically predict at-risk students [1], student performance [2], and early drop-outs [3]. The early prediction of student performance will help educational institutions and instructors to take corrective measures at different levels by providing the required remedial material and support to students predicted as low performers.

Prior studies generally predict student performance using traditional machine learning. In these studies, authors characterize students based on features extracted from demographic, socioeconomic, academic, and interaction information. The extracted features are used to train machine learning models such as random forest, decision tree, linear regression, and support vector machine to predict student performance [2,4]. However, feature engineering is time-consuming, tedious, and manual. Advancements in neural networks have promising results in diverse NLP applications, namely, sentiment analysis, speech recognition, and machine translation. The researchers also utilize deep learning in educational analytics problems, namely student performance prediction [2,5] and early dropout prediction [6,7]. However, it is largely unexplored, whereas existing studies use feature engineering for student characterization. In [2], authors define a list of 54 student attributes and select the top 30 using singular value decomposition. The selected attributes are passed through a dense artificial neural network (ANN), constituting three hidden layers for learning and predicting student performance. Ramanathan and Thangavel [8] apply the steepest gradient Minkowski sommon to map for attributes selection, which further passes through a dense neural network consisting of long short-term memory (LSTM) network for student performance prediction. The researchers have utilized the advancement in deep learning and presented models to predict various aspects of student academic success [9–12]. In [9], authors select the significant feature from the OULA dataset using the butterfly fitness function and further train the deep neural network to predict student performance. Likewise, existing approaches generally introduce hand-crafted features and pass them to an ANN, recurrent neural network (RNN), or a straightforward network. Therefore, existing literature on one of the most widely used benchmark datasets in learning analytics, OULA, utilizes either classical machine learning or deep neural networks with multiple dense layers to predict student performance as a binary classification problem. These approaches generally apply techniques to select significant features and further train models over these for student performance prediction. To the best of our knowledge, we are the first ones to model daily student interaction with the learning platform as a 2d matrix representing their diurnal rhythmic behavior. We also model student interaction with 20 types of VLE materials to capture their interaction information during the three periods: before and after course commencement and finally, overall interaction. Therefore, in contrast to existing studies evaluated over the OULA dataset, MultIFAR models the multidimensional information representing different aspects of student behavior using attentional LSTM and CNN networks and further infuses them to learn the aggregate student representation. It is further passed through a dense layer to predict the student’s academic performance.

This study introduces a deep neural network model, MultiFAR, developed to analyze diverse categories of student information, including demographic, academic performance, and VLE interaction data. The model is trained to accurately predict student performance in a course by leveraging the multifaceted dataset. In the model, CNN extracts spatial and temporal features from image datasets, utilizing convolutional and max-pooling layers. It enables the model to capture intricate patterns and correlations within the data, paving the way for more accurate predictions. However, researchers also use CNN in text-processing problems because of its ability to extract high-level features [13]. In this paper, we model clickstream information, representing student interaction with VLE as diurnal behavior to capture their inter- and intra-day interactions. Further, we apply six-layer CNN over it to extract high-level interaction features. The clickstream information is sequential, so we apply BiLSTM to learn temporal features, further augmented with student assessment information. The BiLSTM is a recurrent neural network architecture for sequential information, incorporating context for a particular feature in the forward and backward directions. The existing literature reveals that demographic and socio-economic factors also affect student performance. Therefore, we also create a 9-dimensional demographic vector and pass it through a BiLSTM network to include background information. The CNN and BiLSTM architectures have no specified mechanism to handle and weigh significant features. We have introduced an attention layer to address this limitation in the CNN and BiLSTM architectures. It allows the model to focus more on the crucial features. Subsequently, the extracted features from all three components are combined and processed through a dense layer. Following this, a model utilizes a softmax layer to predict the student performance. In conclusion, we summarize the main contributions in the following points:

- Jointly models and infuses multidimensional information in the form of *demographic*, *assessment*, and *interaction* information to the devised deep architecture.
- Presents a novel *attention-driven* Covolutional and BiLSTM network-based deep model to process and learn student representation using the multidimensional information.
- Conduct an in-depth comparative evaluation using a benchmark dataset to investigate the model efficacy. Also, we carried out a detailed component ablation analysis to meticulously assess the influence of each behavioral component.
- Evaluate the model for early prediction of student performance to facilitate decision-makers and institutions for early intervention.

## 1 Literature survey

Numerous methodologies exist in the literature for analyzing and modeling the diverse facets of student accomplishment in virtual learning environments. These aspects include dropout prediction [6,14,15], at-risk student prediction [1], performance prediction [2,16], and identification of slow learners [17]. We can classify the existing approaches in educational data mining into two broad categories: predictive analytics and learning analytics [18]. The predictive analytics approaches aim to predict student performance, failure rate, and at-risk students [19]. In learning analytics, authors collect and analyze student information to gain insights into their learning behavior, cognition, and interaction experience to improve their attainment. The existing literature has review articles that provide detailed discussions of the evolution of educational data mining [18–20]. In classical feature engineering-based machine learning, researchers have used categories of features, namely, socio-demographic, assessment, VLE-interaction, and academic history for student performance prediction [21,22]. In these approaches, researchers have used different machine learning models like Naive Bayes, NBTree, J48, SVM, kNN, Random Forest, and XGBoost for predicting student performance [23–27].

The classical machine learning-based approaches characterize students using a set of manually devised features, which is time-consuming and tedious. Recently, researchers have observed the rapid use of deep learning, CNN, and RNN algorithms in diverse applications of NLP and computer vision [28–30]. The researchers have presented deep learning approaches to investigate various aspects of student performance, namely, drop-out prediction [6,31], wheel-spinning problem [32], and knowledge retention [5]. Similarly, they have presented deep learning approaches to predict student grades in a course/program [2,8,16]. In an early approach, Alam et al. [33] defined features based on the user’s activity and trained a deep belief network along with five other classical machine learning models to classify students into low, medium, and high rankers. Raga and Raga [34] modeled the student activities in a Moodle using a simple feed-forward neural network to predict their performance in mid-term and final exams early. The author used the activity log of 885 students and found that data from an extended period positively impacts the model accuracy. In [35], authors presented course-specific Multilayer Perceptron and RNN-based models to predict grades in future courses employing the student’s previous grades. Waheed et al. [2] first handcrafted a 54 feature extracted from students’ demographic, assessment performance, and click-stream information. They applied SVD for dimensionality reduction and modeled it using a simple artificial neural network. They extended the presented approach for early prediction of at-risk and high-performing students.

In a study [16], Karimi et al. modeled the relationship between the student and course as a knowledge graph. They applied the graph convolution network to train the student and course embeddings. They also employed LSTM to encode student behavioral data and then combined it with student and course embeddings to forecast student performance in a course. In their research, Tao et al. [36] utilized a graph convolutional network, a neural architecture for graph data, to predict student performance. Ramanathan and Thangavel (2021) employed a stacked LSTM-based deep learning model to forecast student performance in a course. These approaches model the sequential nature of student socio-economic and clickstream data generated through interaction with VLE using recurrent neural networks. Recently, researchers exploited the advancement in deep learning and presented models to predict various aspects of student academic success [9–12,37]. In a recent study, Wang et al. [11] applied collaborative filtering to preprocess the dataset and then used metadata clustering to resolve the imbalance of academic features. It further trains the XGBoost-enhanced model to predict student performance. In another study [9], authors used the butterfly fitness function to select significant features from the OULA dataset and further trained the deep neural network to predict student performance. In another approach, Junejo et al. [38] presented a convolution and LSTM-based model to predict student grades. They processed a Jordanian dataset, extracted 46 features, and converted it as single-channel data to pass it to the model. Researchers have also presented multiclass classification approaches over the OULA dataset to predict the four categories of students rather than modeling them as a binary class problem [39,40]. This manuscript focuses on binary classification, although we also evaluated the MultiFAR for multiclass classification.

## 2 Proposed methodology

**Fig 1:**
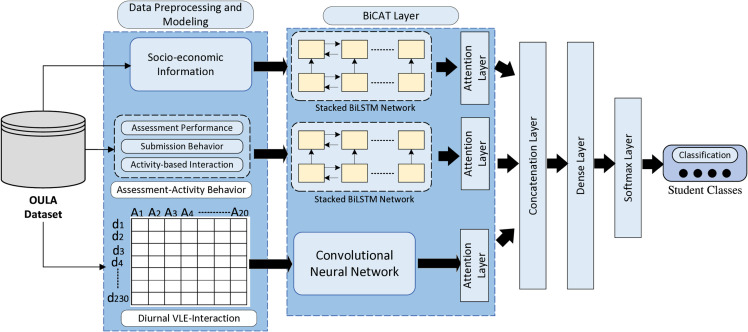
Architecture of MultiFAR for attention-driven representation learning.

This section outlines the workflow of the proposed methodology for predicting student performance. Fig 1 provides the graphical representation of MultiFAR architecture. The following subsections present a precise and comprehensive description of each layer of the model.

### 2.1 Data preprocessing and modeling

We will harness input data to model three main behavioral characteristics of students. These are described briefly in the following paragraphs.

#### 2.1.1 Socio-economic information.

In the existing studies, researchers have demonstrated that socio-economic and demographic information of students influenced their output in a course [2,25]. The socio-economic behavior, represented using 𝒮, has 9 attributes, namely, *gender*, *region*, and *highest education* to characterize the students in terms of socio-economic information.

#### 2.1.2 Assessment-interaction behavior.

This representation is based on the following three behavioral representations, which are described in the following paragraphs:

#### Assessment performance.

The student’s performance in internal assessment of a course is generally considered an early indicator of their final performance. The Open University in the OULA dataset evaluates students considering three methods: tutor-mediated assessment (TMA), computer-mediated assessment (CMA), and final exam. In a course, an instructor conducts assessments through multiple tutor-mediated and computer-mediated Finally, students are assessed using a final exam. Each assessment exam has a different relative weight. We include only tutor-mediated evaluations and the final exam because the weight of computer-mediated exams is lower and insignificant than the TMA and final exam. We include student performance only in the first five TMA and final exam to characterize their performance. Finally, a student’s performance is represented using a set of 6 attributes. We consider only five TMA assessments because most courses have less than five assessment exams.

#### Submission behavior.

In a course, instructors provide assignments to students with a deadline. Its submission behavior also reflects student punctuality and sincerity. Therefore, we model the submission behavior of a student considering the deadline. In the OULA dataset, most courses have five assignments. As a result, we represent the student submission behavior using a set of 5 attributes.

#### Aggregate activity-interaction.

The click-stream information representing student interaction with a VLE is vital for predicting student performance in a course. In the OULA dataset, a student can access 20 types of resources over the VLE. Students can assess these resources even before the course commencement. Therefore, we extract student interaction with 20 types of VLE resources in three periods: (i) before the course commencement, (ii) after the course commencement, and (iii) overall. We compute the sum of all interactions for each resource type for the three periods. Finally, the activity-interaction behavior of a student is represented using a 60-d feature.

Finally, MultiFAR concatenates three behavior vectors to represent the student assessment-interaction-based behavior representation 𝒜, using a 71-d feature vector.

#### 2.1.3 Diurnal VLE-interaction.

The click-stream information representing student interaction with resources over VLE is vital in finding regularity in the diurnal interaction pattern. It contains the number of interactions and the underlying date with a particular resource for each student. To observe the evolution of student interaction behavior with the available resources, we count everyday student interactions with each resource. Similarly, the model computes the sum of interactions with each of the 20 resources every day. In the OULA dataset, students generally start interaction one month before the commencement of a course up to a maximum of 9 months. We model the student interaction behavior from one month before the course commencement to 200 days, considering only 200 days, although the course duration is between 235 to 270 days. It is because, before selection, we conducted an analysis of all the modules and found that there was hardly any interaction data after 200 days. After 200 days, most of the values were 0. So, we consider only the first 200 days of data to avoid sparseness. Finally, we represent the diurnal VLE-interaction, represented using ℐ, with 20 different activities using a matrix ${ℳ}^{d\times a}$, where *d* and *a* represent the number of days having interaction and the number of activities, respectively.

### 2.2 BiCAT layer

This section presents a detailed functional description of the proposed model, consisting of a stacked BiLSTM network, convolutional neural network, and attention layers. A brief description of these layers, components, and their functionality is presented in the following subsections.

#### 2.2.1 Stacked BiLSTM network.

The socio-economic and assessment-based representations are passed to a separate 2-layer stacked BiLSTM network. The BiLSTM, short for Bidirectional Long Short-Term Memory, represents an evolution of the LSTM cell, which is a type of RNN architecture designed to handle sequential data and model temporal dynamic behavior [41]. The fundamental advantage of BiLSTM over LSTM lies in its ability to address the *vanishing gradient* problem more effectively. The LSTM architecture consists of memory blocks that are created from memory cells, and empower it to make informed decisions about what to retain and what to discard. This feature equips LSTM networks with the capability to learn and retain long-range contextual information, making them adept at capturing complex sequential patterns. An LSTM cell is composed of three fundamental components known as digital gates: the input gate *i*_*t*_, the forget gate *f*_*t*_, and the output gate *o*_*t*_ that together with a memory cell state *c*_*t*_ play a crucial role in the functioning of the LSTM network. The *i*_*t*_ at time stamp *t* performs the pivotal function of regulating the flow of information into the cell, and it also updates the cell’s state to a new value using a specific mathematical equation (see Eq 1). Concurrently, the forget gate is responsible for determining the extent to which information is to be discarded at time *t*, employing its distinct equation (see Eq 2). It calculates the candidate cell value $\tilde{C_{t}}$ using Eq 3. Then, it computes the current cell state value *C*_*t*_ using 4. The output gate *o*_*t*_ is determined by 5, and in the last, it computes the final value *h*_*t*_ of the LSTM cell at time *t* using 6. In the given set of equations, the symbols *W* and *b* correspond to the weight and bias vectors, respectively. The σ() denotes the sigmoid function, which introduces non-linearity in the neural network. Similarly, tanh signifies the hyperbolic tangent function. Furthermore, ⊗ denotes the element-wise multiplication operation applied component-wise to the vectors or matrices involved in the computation.

$$i_{t}=\sigma (W_{i}\cdot [h_{t-1},x_{t}]+b_{i})$$ (1)

$$f_{t}=\sigma (W_{f}\cdot [h_{t-1},x_{t}]+b_{f})$$ (2)

$$\tilde{C_{t}}=\tanh(W_{C}\cdot [h_{t-1},x_{t}]+b_{C})$$ (3)

$$C_{t}=f_{t}⊗C_{t-1}+i_{t}⊗\tilde{C_{t}}$$ (4)

$$o_{t}=\sigma (W_{o}\cdot [h_{t-1},x_{t}]+b_{o})$$ (5)

$$h_{t}=o_{t}⊗\tanh(C_{t})$$ (6)

This study opted to use BiLSTM instead of regular LSTM to empower the model to capture both forward and backward contexts. The BiLSTM achieves this by employing a pair of LSTM networks: one captures future context by processing the representation vector from left to right (forward LSTM), and the second LSTM captures historical context by processing the representation vector from right to left (backward LSTM). This results in the generation of two hidden states, $\vec{h_{t}}$ and $\overset{\leftarrow }{h_{t}}$, as demonstrated mathematically in Eqs 7 and 8, respectively. The BiLSTM combines $\vec{h_{t}}$ and $\overset{\leftarrow }{h_{t}}$ to learn *h*_*t*_ 9. The deep recurrent neural network is known for its ability to learn low-level feature representation. It also accommodates greater model complexity, as detailed in Pascanu’s work [42]. To this end, the model employs a 2-layer stacked BiLSTM to capture better low-level feature representation. We then direct the two resulting feature vectors into separate attention layers to further enhance their significance in the model.

$${\vec{h}}_{i}=\vec{LSTM}(f_{i})$$ (7)

$${\overset{\leftarrow }{h}}_{i}=\overset{\leftarrow }{LSTM}(f_{i})$$ (8)

$$h_{i}=[{\vec{h}}_{i},{\overset{\leftarrow }{h}}_{i}]$$ (9)

#### 2.2.2 Convolutional neural network.

The socio-economic and assessment-based representations are injected into separate stacked-BilSTM networks because these are sequential information. However, BiLSTM cannot extract vital local features. To observe student diurnal interaction behavior with VLE, we pass the daily interaction information across 20 activities into a convolutional neural network. CNN is a type of neural architecture primarily employed to process grid-format data, such as images and text arranged in a matrix [43]. One of the main advantages of CNN is its ability to effectively extract local and position-invariant features from the data [13]. CNN performs two operations - *convolution* and *pooling* to extract relevant features from the grid-shaped data. In these, *convolution* uses filters in matrix form over the input representation and extracts high-level relevant features called feature-map. These feature-map capture relevant patterns and structures present in the input data. The *pooling* operation down-sample the *feature-map* by selecting critical features and reducing the spatial dimensions. The researchers originally designed the convolutional network to process and analyze image datasets. Later, it gained widespread adoption in various natural language processing and text classification tasks because of its ability to capture intricate patterns and features from textual data. In deep CNNs, the higher layers build over the lower layers capture rich and complex features [44]. To this end, we implemented a six-layer CNNs. Once the CNNs have extracted the feature vector, an attention layer further processes it. This layer dynamically assigns weights to each feature, evaluating their discriminative power and relevance. It ensures that the most influential features are given priority in predicting student performance, resulting in improved model performance.

### 2.3 Attention and concatenation layer

At this stage, the model infuses the three feature vectors learned through temporal BiLSTM and spatial CNN. These feature vectors are then fed into an attention layer to determine their respective attention scores, with higher scores assigned to significant features and lower scores given to less important ones. Suppose *h*_*f*_ is the learned feature vector of *f*, first, it passes this representation to a dense layer to learn the output representation $h_{f}^{\prime }$ (see Eq 10). Furthermore, the attention layer computes the similarity between $h_{f}^{\prime }$ and $v_{f}$, a random vector. Finally, the attention layer calculates the attention score using a softmax function as defined mathematically in Eq 11. The vertex tensor $v_{f}$ is initially randomized and then learned with other parameters during the training process, as described in [45].

$$h_{f}^{\prime }=\tanh(wh_{f}+b)$$ (10)

$$\alpha_{f}=\frac{exp(h_{f}^{\prime }v_{f})}{\sum_{f}exp(h_{f}^{\prime }v_{f})}$$ (11)

Finally, the model takes the attention-based feature representation of three feature vectors and combines them into a single feature representation. This combined representation is then input into a dense layer, which processes the information and produces an output. The output from the linear layer is further fed into a softmax layer, which classifies the student’s performance based on the processed features.

## 3 Experimental setup and evaluation results

This section assesses the MultiFAR model over the popular Open University Learning Analytics dataset. The dataset description encompasses its key attributes, including size, feature categories, and other details. Following this, a thorough performance analysis of the model is conducted, which involves assessing its accuracy, precision, recall, loss, and AUC. Moreover, we conducted a detailed comparative evaluation to compare the MultiFAR with state-of-the-art (SOTA) approaches, transformer-based models, and baseline methods. We also evaluated MultiFAR for multiclass prediction and investigated the balanced version of the OULA dataset. Furthermore, we conduct a component ablation analysis to uncover the influence of each component of MultiFAR over its performance. It will result in valuable insights regarding the importance of each neural architecture of MultiFAR.

### 3.1 Dataset description and evaluation metrics

We evaluate MultiFAR over the widely used educational data mining dataset - OULA [46]. It contains data on student performance in internal assessments and final exams, demographic information, and interaction with various learning materials over the VLE in the form of a clickstream for 32593 students. The authors collected data over 9 months from 2014 to 2015 for seven courses. In the dataset, courses are called modules, and teaching semesters are presentations. The dataset has 7 Excel files containing all the information. Based on the mark in a course, students are divided into four categories, namely, pass, fail, distinction, and withdrawn having 12361, 7052, 3024, and 10156 students, respectively.

We investigate the effectiveness of the presented model using four metrics: *accuracy*, *precision*, *recall*, *loss*, and *AUC*. In this study, we convert predicting student performance in a course into four binary classification problems. For example, in the pass/fail prediction problem, pass is a positive class, and fail is a negative class. Accuracy in this context refers to the proportion of correctly predicted grades out of the total students. This measure reflects the ratio of correctly predicted student grades to the total number of students whose grades are classified by the model. Mathematically, it is defined as outlined in Eq 12. It’s important to note that false positives (FP) indicate the number of negative class students incorrectly classified as positive. *Precision* measures the proportion of correctly predicted positive class grades out of all the student grades predicted as positive. Mathematically, Eq 13 defines its calculation. Recall quantifies the ratio of correctly predicted positive class student grades to the total number of positive student grades, as defined in Eq 14. The loss function measures the total difference between the predicted class instances and the actual class instances. It represents the sum of the discrepancies between the predicted and underlying actual class instances. Lastly, AUC, the area under the curve, is a metric used to evaluate the performance of a binary classification model. AUC measures a model’s ability to distinguish between positive and negative classes.

$$\text{accuracy}=\frac{\text{TP}+\text{TN}}{\text{TP}+\text{FP}+\text{TN}+\text{FN}}$$ (12)

$$\text{precision}=\frac{\text{TP}}{\text{TP}+\text{FP}}$$ (13)

$$\text{recall}=\frac{\text{TP}}{\text{TP}+\text{FN}}$$ (14)

### 3.2 Training detail

The MultiFAR utilizes a two-layer stacked BiLSTM to process the sequential information vector. Each layer of the network has 512 memory cells. Moreover, the model incorporates a six-layer convolutional network for representation learning from interaction data. The first three convolutional layers have 512 filters with a kernel size of 2×2. Thereafter, the MultiFAR applies a max-polling operation of 3×1 to the feature-map. The last three convolution layers also have 512 filters, but kernel size is 2×1. Later, a max pooling operation of 2×1 is applied on the resulting feature-map. The MultiFAR further takes three separate feature vectors. To prevent overfitting, a dropout of 0.5 is applied. This layer forwards the output to a sigmoid layer with 2 neurons for classification. Considering optimization, the model applies categorical cross-entropy to minimize the loss. The MultiFAR uses the Adam optimizer with a learning rate of 0.001. In the evaluation, the MultiFAR is trained over five-fold cross-validation. We used this evaluation strategy in every experiment in this manuscript. This approach ensures a robust assessment of the MultiFAR performance.

### 3.3 Performance evaluation results

We thoroughly assess the effectiveness of the MultiFAR over the OULA dataset using five standard evaluation metrics: *accuracy*, *precision*, *recall*, *loss*, and *AUC*. We investigate the efficacy of MultiFAR with three state-of-the-art (SOTA) models: [2,47,48] and two transformer-based models [49,50]. Also, we performed the comparison with five baseline models constructed from MultiFAR. Table 1 presents the experimental results of MultiFAR along with SOTA models [2,47,48], transformer-based models [49,50], and baselines. All the compared models have used the OULA dataset to evaluate their efficacy and used the same hyperparameter adjustment. It’s important to note that the OULA dataset categorizes the student grades in distinction, pass, fail, and withdrawn categories. This paper models predicting student grades as a binary classification problem for different pairs of student grade categories. The first two problems predict at-risk students, whereas the last two predict high-performers. A brief discussion of the four classification problems is given in the following subsections:

**Table 1:** Comparative results of MultiFAR, SOTA, transformer-based models, and baseline approaches.

Approach	Pass/Fail					Pass/Withdrawn					Distinction/Fail					Distinction/Pass				
	Acc	Prec	Recall	Loss	AUC	Acc	Prec	Recall	Loss	AUC	Acc	Prec	Recall	Loss	AUC	Acc	Prec	Recall	Loss	AUC
MultiFAR	**89.57**	92.57	71.65	**0.286**	**0.961**	**97.12**	94.18	93.49	**0.114**	0.987	**92.06**	**93.28**	94.10	**0.224**	**0.949**	80.31	80.06	**97.86**	0.575	**0.925**
Waheed et al. [2]	84.48	86.00	61.70	0.385	0.916	94.70	94.00	91.70	0.136	0.965	86.40	78.00	76.00	0.295	0.859	80.54	69.00	82.00	0.591	0.834
Song et al. [47]	86.21	90.14	71.17	0.321	0.937	96.32	**94.45**	92.05	0.131	0.983	88.87	89.41	90.23	0.267	0.935	81.25	79.15	95.49	**0.495**	0.915
Waheed et al [48]	85.12	87.56	65.32	0.348	0.933	91.52	90.19	89.62	0.148	0.968	83.31	79.14	80.55	0.327	0.872	79.71	77.63	85.92	0.634	0.883
Huang et al [49]	87.94	86.75	77.88	0.301	0.913	95.85	93.10	94.10	0.201	**0.989**	91.11	87.57	94.56	0.287	0.944	83.17	**83.76**	88.05	0.604	0.901
Cholakov and Kolev [50]	88.24	**93.65**	76.54	0.291	0.922	96.11	93.54	93.91	0.158	0.968	90.31	88.41	93.39	0.229	0.929	**81.47**	78.36	86.29	0.643	0.898
Simple Model	88.93	86.00	74.92	0.691	0.945	95.97	83.08	**95.10**	0.349	0.968	88.24	91.99	88.17	0.922	0.938	78.55	81.16	94.43	0.895	0.864
BiLSTM_CNN	87.41	77.00	**79.14**	0.451	0.934	95.30	86.50	93.05	0.208	0.956	88.89	82.33	95.44	0.394	0.929	79.40	81.00	94.09	0.533	0.891
ANN	85.67	75.71	76.63	0.897	0.899	95.13	89.36	94.19	0.567	0.959	84.75	85.36	**96.07**	0.759	0.910	78.66	78.32	88.78	0.496	0.869
AttBiLSTM	84.31	82.80	79.06	0.388	0.923	91.11	92.10	89.29	0.243	0.953	81.40	81.84	80.61	0.3644	0.897	80.25	81.28	80.37	0.668	0.864
AttCNN	66.06	47.46	53.80	0.560	0.780	70.97	55.66	63.89	0.526	0.824	73.06	74.42	67.51	0.558	0.829	76.21	75.55	75.96	0.5512	0.858

#### 3.3.1 Prediction of at-risk students.

It is crucial to identify and provide support to at-risk students in a course. In this paper, MultiFAR predicts two categories of at-risk students: those who failed and those who withdrew from the course. We transformed the task of predicting at-risk students into a binary classification problem consisting of two cases. Firstly, we predicted students at risk of failure alongside successful students, termed as the pass/fail problem. Secondly, we predicted students who might withdraw from a course alongside successful students, known as the pass/withdrawn problem. In both cases, pass and distinction students are combined in a single group called pass having 15385 students. The experimental results for the two categories of problems are present in 2–9 columns of the third row of Table 1. The MultiFAR model for pass/fail and pass/withdrawn show an accuracy of 89.57 and 97.12, respectively. Similarly, we can observe the results considering *precision*, *recall*, *loss*, and *AUC* for the two categories of problem.

#### 3.3.2 Prediction of high-performers.

The MultiFAR predicts high-performers (distinction) along with pass students and those who are at risk of failure. It will model these two combinations as separate binary classification problems: distinction/fail and distinction/pass. The MultiFAR in distinction/pass predicts a student will either pass only or get the distinction. In distinction/fail, the model predicts that either a student will achieve a distinction or fail. The last ten columns of Table 1 present the evaluation results corresponding to these two problems for MultiFAR along with SOTA and baseline approaches. The MultiFAR accuracy for distinction/fail and distinction/pass problems is 92.06 and 80.31, respectively. MultiFAR performs poorly in distinction/pass category due to significant data imbalance between the *distinction* and *pass* students in a ratio of 1:4.

### 3.4 Comparative evaluation

To investigate the effectiveness of MultiFAR, we conduct a comprehensive comparison with three SOTA [2,47,48], two transformer-based models [49,50] and five baseline approaches. The comparison methods are briefly outlined in the following paragraphs:

[2] The authors introduced 54 features based on socio-economic, assessment, and VLE-interaction information. They applied singular value decomposition to select the top 30 features and passed it to a simple artificial neural network with 3 hidden layers for four categories of binary classification problems. They also evaluated the model using the OULA dataset.
[47] This study presents a sequential engagement-based academic performance prediction network (SEPN). It includes two main components: an engagement detector and a sequential predictor. The engagement detector has CNN to track student engagement patterns based on their daily activities. The sequential predictor uses LSTM to learn the interaction from the engagement feature spaces and demographic features.
[48] The authors investigated the prediction of student performance in a self-paced environment. To this end, they investigated the performance of an LSTM model in predicting students at risk of failure in a self-paced course. They also evaluated this study over the OULA dataset.
Huang [49]: To evaluate the performance of MultiFAR against the transformer-based models, we compared it with two models for tabular data. The first, TabTransformer, is a transformer-based deep model suitable for tabular data. It utilizes self-attention mechanisms to model the dependencies between categorical and numerical features. Its ability to create contextual embeddings for categorical information makes it suitable for the OULA dataset.
Cholakov [50]: This model an extension of the TabTransformer model, having integrated gated multilayer perceptron, which enhances feature selection and interaction modeling. This model is suitable for situations where the datasets are noisy and incomplete. Therefore, it is suitable for OULA and educational datasets as student and interaction information is generally missing or incomplete. It balances complex and simple transformations easily to model student behavior for predicting their performance.
Simple Model The MultiFAR model uses stacked BiLSTM and deep CNN layers. In this baseline, we use a single BiLSTM and CNN layer to observe the impact of multiple BiLSTM and CNN layers on the model performance.
BiLSTM_CNN The proposed model incorporates an attention mechanism to emphasize relevant features, allowing a more nuanced and focused approach to analyzing the student information. To fully comprehend the influence of attention within the model, we analyzed the removal of attention layers. By executing the model without these attention layers, we aimed to assess the impact on the model’s ability to predict student performance.
ANN This baseline concatenates the socio-economic, assessment-activity, and VLE-interaction feature vectors into a 4679 dimensional vector. This vector is given to a deep artificial neural network with three hidden layers having 50, 30, and 15 neurons.
AttBiLSTM In the third baseline, we use the stacked BiSTM network for representation learning of socio-economic, assessment, and activity-based feature vectors. Later, the model injects trained vectors into attention layers for student performance prediction.
AttCNN This baseline passes the diurnal VLE interaction representation into an attention-driven CNN for predicting student performance.

**Fig 2:**
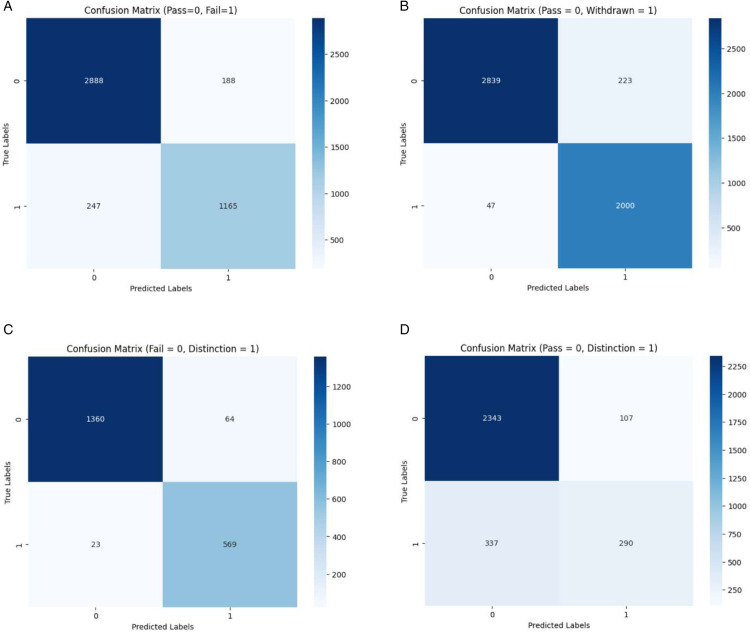
Confusion matrix for the imbalanced dataset.

**Fig 3:**
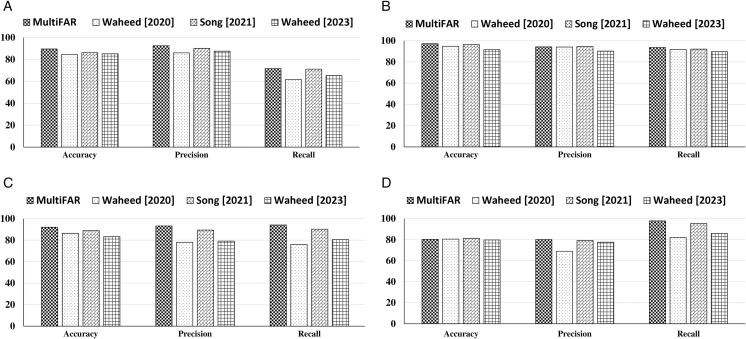
A graphical representation of comparative results of MultiFAR with SOTA and baseline approaches.

Table 1 presents the evaluation results of the MultiFAR compared to SOTA and baselines models considering the *accuracy*, *precision*, *recall*, *loss*, and *AUC*. It reveals that MultiFAR outperforms the three SOTA models [2,47,48] in all cases except three. However, the performance difference in these cases is comparative and not significantly different. The investigation of MultiFAR results with two transformer-based models reveals that MultiFAR outperforms these two models except in nine instances. These transformer-based models show comparative performance but don’t beat MultiFAR. The results show that the MultiFAR exhibits improvement over SOTA and transformer-based models. A simplified version of the MultiFAR called simple model, performs best among the baseline approaches. AttCNN, which models using only the interaction component, shows the worst performance. In Table 1, the best-performing approach considering each metric is shown in bold font. Fig 2 presents the confusion matrix representation for all the four classification task. Also, a graphical visualization of comparative performance with SOTA considering *accuracy*, *precision*, *recall* is shown using Fig 3.

#### 3.4.1 Evaluation over balanced dataset.

The benchmark dataset, OULA, used in this paper is unbalanced. In the real world, generally, fewer students withdraw from a course or fail a course. However, we evaluated the MultIFAR efficacy with the SOTA and baseline models over the balanced datasets to further investigate the potency of MultIFAR under a balanced dataset scenario. To this end, to balance the dataset for a particular problem, say, pass/fail, we first select the minimum number of instances from these two categories. Then, we randomly select the same number of instances from the second class. We don’t oversample because they may not reflect the real-world distribution of student categories. The experiment with the MultIFAR, SOTA, and baseline models is performed with the earlier parameter settings. We conducted the evaluation considering the *accuracy*, *precision*, *recall*, *loss*, and *AUC*. The experiment is performed for all four categories of problems: pass/fail, pass/withdrawn, distinction/fail, and distinction/pass. The underlying results considering the evaluation metrics along the SOTA and baselines are presented in Table 2.

**Table 2:** Comparative results of MultiFAR with SOTA and baseline approaches over balanced dataset.

Approach	Pass/Fail					Pass/Withdrawn					Distinction/Fail					Distinction/Pass				
	Acc	Prec	Recall	Loss	AUC	Acc	Prec	Recall	Loss	AUC	Acc	Prec	Recall	Loss	AUC	Acc	Prec	Recall	Loss	AUC
MultiFAR	**94.29**	**94.37**	94.48	**0.134**	**0.971**	96.32	**97.22**	97.43	0.126	**0.987**	**95.27**	**96.13**	93.85	**0.086**	0.977	**98.76**	**99.64**	98.48	**0.042**	**0.997**
Waheed et al. [2]	90.84	89.72	83.60	0.234	0.932	95.34	96.61	94.01	0.158	0.979	94.30	93.08	92.56	0.121	0.961	92.54	89.61	91.41	0.145	0.969
Song et al. [47]	91.32	92.14	89.78	0.249	0.917	95.89	97.31	94.46	0.138	0.979	93.87	95.03	92.51	0.098	0.955	96.54	97.37	97.01	0.067	0.979
Waheed et al [48]	92.36	91.44	89.99	0.296	0.956	95.92	95.88	96.79	0.133	0.970	93.88	94.07	92.19	0.102	0.961	93.16	95.31	96.09	0.072	0.973
Huang et al [49]	93.11	90.72	91.51	0.179	0.948	95.97	95.96	**97.66**	0.145	**0.980**	94.89	95.75	92.64	0.095	0.964	95.53	94.56	96.43	0.077	0.983
Cholakov and Kolev [50]	94.03	93.21	91.71	0.151	0.963	**97.22**	95,99	97.18	**0.119**	0.984	94.83	94.27	**94.18**	0.110	0.965	97.12	97.34	**98.89**	0.053	0.988
Simple Model	94.04	93.88	94.10	0.150	96.14	95.76	93.69	95.84	0.136	96.88	93.54	93.62	94.01	0.122	0.955	95.34	93.45	94.89	0.193	97.63
BiLSTM_CNN	92.73	89.47	92.12	0.194	91.36	93.30	92.45	91.15	0.148	96.54	90.80	89.65	91.10	0.196	96.54	92.21	90.24	93.01	0.133	95.38
ANN	81.46	81.24	81.79	0.542	87.68	95.22	96.42	96.11	0.131	97.41	88.57	86.36	89.30	0.234	91.32	91.56	93.23	89.87	0.204	96.88
AttBiLSTM	87.24	87.82	86.45	0.314	93.57	94.36	93.38	95.35	0.153	96.63	85.53	84.48	86.16	0.302	93.43	96.71	93.69	97.11	0.119	98.01
AttCNN	94.15	93.53	**95.47**	0.484	97.96	95.47	95.71	95.27	0.146	97.57	90.60	91.24	90.15	0.198	94.52	95.12	96.15	97.69	0.251	97.45

The investigation of Table 2 results over the balanced dataset reveals that results over the balanced dataset are improved compared to the original dataset. The performance of the SOTA and baseline models also shows improved performance. However, they are behind the MultiFAR considering all metrics except a few instances. Among the baseline, AttCNN shows the best result and even outperformed our model for pass/fall considering recall. Another interesting observation is that MultiFAR performs best over the distinction/fail and distinction/pass, where the baseline and SOTA models fail to show comparative performance. This may be due to the small number of instances in these datasets. In Table 2, the best-performing approach considering each metric is shown in bold font.

### 3.5 Analysis of impact of deep neural components

In the comparative evaluation, we constructed baselines by removing neural components from the MultiFAR model. For example, the BiLSTM_CNN baseline is created by excluding the attention mechanism from MultiFAR. This section thoroughly investigates the impact of fundamental architectures included in the MultiFAR, and Tables 1 and 2 presents the underlying results. This investigation finds a comprehensive understanding of the influence of each neural component and its settings on the grade classification performance of MultiFAR. Upon analysis, we found that the MultiFAR consistently performs better than the baseline Simple Model in all cases except for three instances. It justifies the inclusion of stacked BiLSTM and deep CNN in the proposed MultiFAR model and shows that it improves the MultiFAR performance. Also, baseline BiLSTM_CNN performs lower than the MultiFAR in all cases. It establishes that the attention mechanism in the MultiFAR positively impacts its performance. Also, to investigate the efficacy of BiLSTM, we constructed the AttCNN baseline, whereas to observe the impact of multi-convolutional layers, we created AttBiLSTM. Comparatively poor results of these baseline models also justify using stacked BiLSTM and multi-convolutional layers with the attention mechanism. In the ANN baseline, we concatenate all three behavioral components into a 4679-d vector. It also shows a pattern of subpar performance. Consequently, the outcomes obtained from numerous established baseline approaches underscore the effectiveness of MultiFAR and its constituent elements in addressing diverse student performance prediction tasks.

### 3.6 Behavior ablation analysis

In this section, we conduct behavior ablation analysis to meticulously assess the impact of each of the three behavioral components: 𝒮, 𝒜, and ℐ on predicting student performance. To investigate the influence of a specific information component, we exclude it from the model. Further, we execute the updated model to study the change in result, representing the impact of that component. For instance, we exclude the assessment vector from the model to study the influence of assessment-related behavior. Following the model updation, we carefully observed and reported its performance. The second row of Table 3 provides the underlying results. We conducted thorough experimentation for the remaining two components and documented our findings. Table 3 presents the result of behavior ablation analysis. The results corresponding to a component showing the highest degradation in the MultiFAR performance are shown in bold font. The table indicates that the II component has the most significant impact on MultiFAR performance for Pass/Fail and Pass/Withdrawn categories across evaluation metrics, except for *recall*. However, it moderately affects the MultiFAR performance for the Distinction/Fail and Distinction/Pass categories. One interesting observation is that excluding the ℐ component improves MultiFAR performance in the Distinction/Pass category, considering *accuracy* and *precision*. The socio-economic component, 𝒮, also shows good discriminating power, especially in the Distinction/Fail and Distinction/Pass tasks. However, it is not as discriminative as the ℐ component. The assessment-interaction component, 𝒜, shows the least impact on the MultiFAR performance. Based on a comprehensive analysis, we can infer that the ℐ component has the most significant adverse impact on the MultiFAR performance. In contrast, the 𝒜 component exhibits the least impact.

**Table 3:** Evaluation results of MultiFAR for component ablation analysis.

Model	Pass/Fail					Pass/Withdrawn					Distinction/Fail					Distinction/Pass				
	Acc	Prec	Recall	Loss	AUC	Acc	Prec	Recall	Loss	AUC	Acc	Prec	Recall	Loss	AUC	Acc	Prec	Recall	Loss	AUC
MultiFAR	89.57	92.57	71.65	0.286	0.961	97.12	94.18	93.49	0.114	0.987	92.06	93.28	94.10	0.224	0.949	80.31	80.06	97.86	0.575	0.925
MultiFAR-𝒮	89.41	87.28	**77.67**	0.2862	0.939	97.06	94.69	92.19	0.103	0.967	83.88	**84.35**	**92.12**	**0.666**	**0.892**	**76.34**	**76.09**	98.48	0.6090	**0.904**
MultiFAR-𝒜	87.10	79.71	82.10	0.3270	0.941	97.16	97.22	**91.81**	0.1150	0.961	91.37	90.86	96.08	0.2417	0.938	79.33	80.84	96.74	0.5747	0.921
MultiFAR-ℐ	**76.36**	**68.41**	84.83	**0.4647**	**0.873**	**90.47**	**77.56**	93.03	**0.2637**	**0.913**	**83.73**	85.16	94.57	0.5683	0.901	81.31	82.48	**95.87**	**0.7461**	0.914

### 3.7 MultiClass classification

The OULA dataset captured information related to four categories of students: distinction, fail, pass, and withdrawn. In the previous subsections, MultiFAR is evaluated for binary classification, as the proposed model is designed for binary classification problems. To further establish the efficacy of MultiFAR, we also investigate its prediction potential for multiclass classification, while preserving the original labels intact. To this end, we implemented MultiFAR and SOTA models as a multiclass model, and the results are presented in Table 4. MultiFAR shows a moderate performance compared to the binary classification. Table 4 also includes the results for SOTA and transformer-based models. We implemented SOTA models using the hyperparameter setting as done in the Sect 3.4. We evaluate the models considering the precision, recall, F1-Score and accuracy. We also present the macro average and weighted average results for precision, recall, and F1-Score. Table 4 results indicate that our model, MultiFAR, performs best considering precision and F1 metrics for both macro average and weighted average. MultiFAR also performs best for the weighted average of recall. On the contrary, SOTA models perform best in terms of accuracy and macro average of recall. Among the models, Waheed et al. [2] perform worst, and transformer-based model by Huang et al. [49] shows the best performance. Among the category-level performances, MultiFAR and SOTA models show the worst and best performances for distinction and withdrawn categories, respectively. The worst performance for the distinction category could be attributed because of class imbalance as distinction class has relatively less number of instances.

**Table 4:** MultiFAR and SOTA results for multiclass classification.

Model →	MultiFAR			Waheed et al [2]			Song et al. [47]			Waheed et al [48]			Huang et al [49]			Cholakov & Kolev [50]		
**Class** ↓	Prec	Recall	F1	Prec	Recall	F1	Prec	Recall	F1	Prec	Recall	F1	Prec	Recall	F1	Prec	Recall	F1
Distinction	75.32	66.34	70.54	64.48	56.87	60.43	71.14	53.45	61.03	63.25	70.80	66.81	61.59	80.24	69.68	60.38	70.20	64.92
Fail	72.19	67.29	69.65	69.29	61.89	65.38	65.55	75.36	70.11	72.51	30.64	43.07	75.87	66.67	70.97	70.46	55.19	61.89
Pass	82.26	70.42	75.88	65.12	50.24	56.72	74.15	64.41	68.93	68.09	60.51	64.07	76.08	62.89	68.85	76.64	64.85	70.25
Withdrawn	75.51	69.07	72.14	66.47	69.52	67.96	72.8	93.9	82.01	74.43	86.69	80.09	80.94	59.68	68.70	89.56	65.24	75.48
Accuracy		69.56			56.64			65.87			59.35			71.55			68.97	
Macro Avg	76.32	68.28	72.07	66.34	59.63	62.80	70.91	71.78	71.34	69.57	62.16	65.65	73.62	67.37	70.35	74.26	63.87	68.67
Weighted Avg	76.11	69.54	72.67	69.23	61.78	65.29	73.49	65.34	69.17	67.24	65.20	66.20	72.59	69.05	70.77	76.04	67.37	71.44

### 3.8 Impacts for educational institutions and policy implications

We could not evaluate the MultiFAR over a real-world scenario due to the unavailability of any recent real-world dataset. However, we demonstrated the efficacy of MultiFAR in diverse conditions, namely, imbalanced and balanced datasets, presenting an early detection version of MultiFAR, investigating its performance with different sets of information through component ablation analysis, and also analyzing the effect of various neural network parameters. From the perspective of institutional practice, the investigated results demonstrated the MultiFAR efficacy and its practical application as an early predictor of student performance. The presented model will facilitate decision-making around early and contextually relevant interventions for students at risk of failure. It will help institutions and policymakers devise strategies and pedagogical policies to provide assistance to at-risk students at crucial junctures and monitor their performance and growth. It will also help institutions to early identify the at-risk students through the analysis of their VLE-engagement. It can also be used by institutions in identifying the meritorious and high-performers so that further assistance can be provided to them so that they do not get distracted.

The insights from this data-driven study demonstrate the possibility of its real-world utility using a reduced dataset. It can also be applied elsewhere as a helpful springboard from which policy stakeholders can formulate pedagogical policies and support guidelines. However, this study has limitations because the dataset is not recent and may not reflect the current scenario. Furthermore, it includes a limited number of demographic attributes, which could have a strong impact on student success. However, MultiFAR results demonstrate that interaction information inhibits strong patterns that can be utilized to predict student performance. Also, fewer demographic attributes provide an opportunity to avoid the ethical problems highlighted by critical authors with the use of personal and demographic data [51].

### 3.9 Ethical aspects

All the experiments in this research are conducted for academic purposes to understand the impact of different categories of information on student performance. The OULA dataset used in the manuscript is a freely available benchmark dataset. So, we don’t need any consent for its usage. The OULA paper [46] describes that the data anonymization was done as per the ethical and privacy requirements of Open University. Therefore, this dataset doesn’t include any private information about students and modules. We didn’t share any information from this dataset with any third party or other researcher and further ensure that we will not share it in the future. Those who need any information can get it directly from the authors of this paper [46]. A piece of detailed information regarding different ethical aspects of this dataset can be found in the original OULA manuscript [46].

## 4 Early prediction of student performance

The early prediction of student performance is crucial for the institution’s decision-making process regarding early intervention. We extended the proposed MultiFAR model for early performance prediction. To achieve this, we divided each module period into four equal quarters - Q1, Q2, Q3, and Q4. The module period is one month from the module start date to the last interaction date. In the early detection approach, we assume the socio-economic behavior 𝒮 like discussed in Sect 2.1.1 because this information for a student is available before the module presentation. Also, the assessment-interaction behavior 𝒜 includes student performance in internal exams and assignment submission behavior, which are generally unavailable in the early days of module presentation. Therefore, we exclude these two behaviors while representing the assessment-interaction behavior. As a result, 𝒜 will now be only 60-d. However, the 60-d 𝒜 vector will be constructed using only the available click-stream up to that quarter. If the length of each quarter is 60 days, then 𝒜 for quarter Q2 will be computed based on click-stream information available up to 120 days. Finally, the diurnal VLE-interaction vector *I* will be created using only the click-stream VLE-interaction information available up to that quarter. Finally, we train the model using student representation for each quarter dataset for the four categories of problems.

### 4.1 Early prediction of at-risk students

The early prediction of at-risk students is paramount for making well-informed decisions. Like Sect 3.3.1, we focus on the early identification of two categories of at-risk students: (i) those who are at risk of academic failure and (ii) those who are at risk of dropping out of course. The early intervention for both groups is essential for providing the necessary support and resources to help them succeed. These two categories of students are potentially at-risk students. We investigate the early prediction of these student grades considering accuracy and loss against those who perform well in the course. This results in two binary classification problems: Pass/Fail and Pass/Withdrawn. We evaluate these two problems over the four quarters of the dataset. The underlying results are given in Table 5. It is evident from the table that our proposed model predicts the two categories of at-risk students with an accuracy of 87.88 and 95.61, respectively, using only 25% of click-stream data and socio-economic information. We can further observe that, generally, the model performance increases as we integrate the information from succeeding quarters. However, one interesting observation is that when we add the third quarter data to the earlier data, model performance decreases in both categories of problems.

**Table 5:** MultiFAR results for early student performance prediction over datasets from different quarters.

Model	Pass/Fail		Pass/Withdrawn		Distinction/Fail		Distinction/Pass	
	Accuracy	Loss	Accuracy	Loss	Accuracy	Loss	Accuracy	Loss
Q1	87.88	0.324	95.61	0.133	83.68	0.427	76.21	0.620
Q2	88.79	0.322	97.36	0.093	84.07	0.386	76.37	0.610
Q3	86.90	0.387	96.89	0.109	82.63	0.515	76.31	0.565
Q4	88.59	0.315	97.10	0.102	87.11	0.342	76.57	0.543

### 4.2 Early prediction of high-performers

The early prediction of potential high-performers can help policymakers focus on such students. To this end, we predict the high performers of a course using the information generated during the early phase of module presentation. Like Sect 3.3.2, we early predict the high performers with fail and pass categories of students. The results for predicting Distinction/Fail and Distinction/Pass grades classification problems are given in the last two columns of Table 5. It includes results for datasets across the four quarters of student information. The table shows that in the case of Distinction/Fail, MultiFAR can predict the high performers with an accuracy of 83.68% using information generated during the quarter of a course period. Also, it increases to 87.11% as we extend the dataset from quarter Q1 to the four quarter data of click-stream and socio-economic information. However, in the case of distinction/pass, our models show considerably good performance with Q1 data, but it doesn’t improve as more quarters data is augmented.

### 4.3 Comparative evaluation

**Fig 4:**
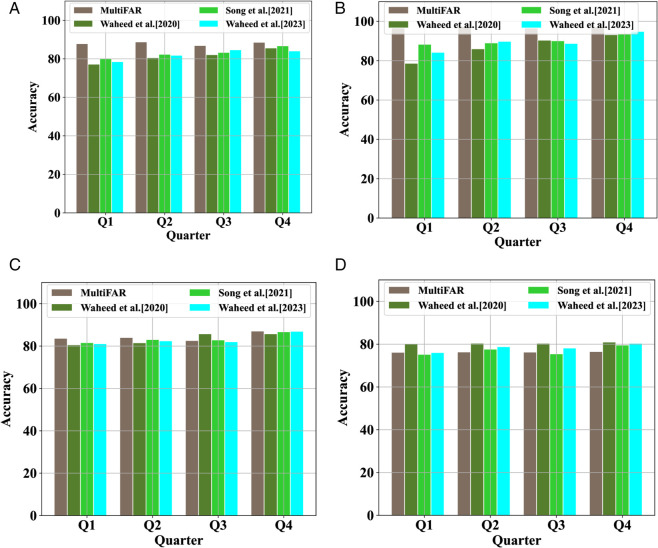
Comparative evaluation results of MultiFAR for four student classification tasks: Pass/Fail, Pass/Withdrawn, Distinction/Fail and Distinction/Pass considering accuracy over the datasets from different quarters.

**Fig 5:**
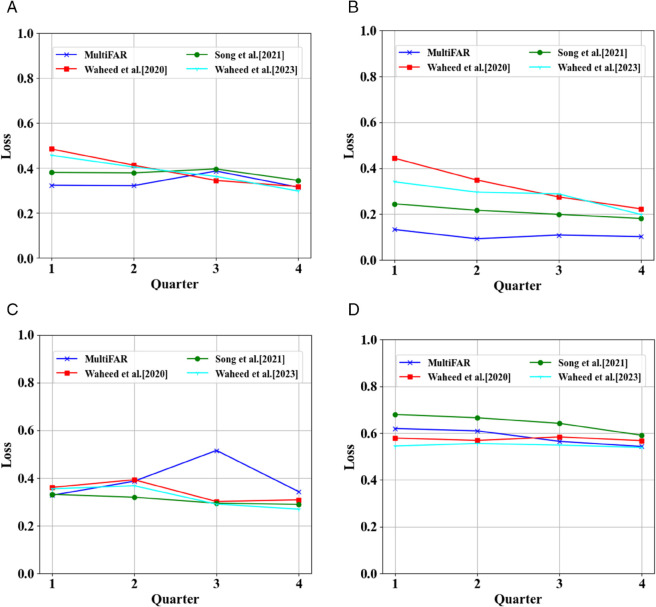
Comparative evaluation results of MultiFAR for four student classification tasks: Pass/Fail, Pass/Withdrawn, Distinction/Fail and Distinction/Pass considering loss over the datasets from different quarters.

We also assessed the comparative evaluation of our early prediction approach with SOTA models [2,47,48] at different quarters of data to classify four categories of students. Fig 4 presents the comparative evaluation of our model with SOTA models considering *accuracy*. We can observe from Fig 4(a) and 4(b) that considering early prediction of at-risk students, MultiFAR significantly outperforms the SOTA models using only Q1 or Q2 datasets that are crucial for decision and policymakers. However, SOTA models show comparative performance as more quarters of data are augmented. However, our model still performs better. For early prediction of high-performer considering distinction/fail classification problem, MultiFAR outperforms SOTA models with an accuracy of 83.68%, using click-stream data generated during the first quarter of the module. Also, when the models are trained corresponding to three-quarters of the dataset, [2] performs better than MultiFAR, though the difference is insignificant. However, our model shows poor performance in the early prediction of high-performers with pass students across all the quarters of the dataset. One interesting observation from Distinction/Pass results is that the performance is nearly consistent over all four quarters of datasets for both MultiFAR and comparison approaches. Similarly, the comparative evaluation considering *loss* over the datasets from different quarters can be found in Fig 5. The careful analysis of the figure reveals that MultiFAR outperforms SOTA models in most cases across various quarters of datasets.

The poor performance of our model considering accuracy and loss in the case of distinction/pass cannot be a case of concern. However, the early prediction of at-risk students is more important than that of high-performers. In this line, our model performs significantly well in the early case with only a quarter of the dataset.

## 5 Concluding remarks and future direction of works

This paper introduced an attention-driven hybrid neural network aimed at predicting the academic performance of students who are at risk as well as who are high-performing students in a course. The students are predicted into four categories: distinction, pass, fail, and withdrawn using multifacet student information. We evaluated the proposed model over a widely recognized benchmark dataset. The experimental analysis reveals that how students interact with the VLE is a good predictor of student’s overall academic success. Our research found that while socio-economic behavior is substantial, it has minimal impact on student performance whereas VLE-interaction information shows the highest impact. We expanded our approach to early predict the students who are at risk of underperforming or dropping out from the course and students who are likely to excel. It will help university decision-makers intervene early and take corrective action. Our expanded model showed good performance in predicting at-risk and dropout students using the data from the first quarter only. In our upcoming research endeavors, we aim to improve our current methodology further to refine our ability to predict student performance. It will involve integrating more comprehensive student feedback data and leveraging cutting-edge natural language processing techniques to gain deeper insights. Furthermore, we are keen to investigate the influence of various VLE activities on student performance, aiming to uncover valuable insights that can inform future educational practices.

## References

[1] Chui KT, Fung DCL, Lytras MD, Lam TM. Predicting at-risk university students in a virtual learning environment via a machine learning algorithm. Computers in Human Behavior. 2020;107(2):1–7.

[2] Waheed H, Hassan S-U, Aljohani NR, Hardman J, Alelyani S, Nawaz R. Predicting academic performance of students from VLE big data using deep learning models. Computers in Human Behavior. 2020;104:106189. doi: 10.1016/j.chb.2019.106189

[3] Santos JL, Klerkx J, Duval E, Gago D, Rodríguez L. Success, activity and drop-outs in MOOCs an exploratory study on the UNED COMA courses. In: Proceedings of the Fourth International Conference on Learning Analytics and Knowledge. 2014. p. 98–102. 10.1145/2567574.2567627

[4] Iatrellis O, Savvas IK, Fitsilis P, Gerogiannis VC. A two-phase machine learning approach for predicting student outcomes. Educ Inf Technol. 2020;26(1):69–88. doi: 10.1007/s10639-020-10260-x

[5] Sharada N, Shashi M, Xiong X. Modeling student knowledge retention using deep learning and random forests. Journal of Engineering and Applied Sciences. 2018;13(6):1347–1353.

[6] Wang W, Yu H, Miao C. Deep model for dropout prediction in MOOCs. In: Proceedings of the 2nd International Conference on Crowd Science and Engineering. 2017. p. 26–32. 10.1145/3126973.3126990

[7] Xing W, Du D. Dropout prediction in MOOCs: using deep learning for personalized intervention. Journal of Educational Computing Research. 2018;57(3):547–70. doi: 10.1177/0735633118757015

[8] Ramanathan K, Thangavel B. Minkowski sommon feature map-based densely connected deep convolution network with LSTM for academic performance prediction. Concurrency and Computation. 2021;33(13). doi: 10.1002/cpe.6244

[9] Parvati V, Belavgi A. Optimized deep network based students performance analysis for college admissions. Multimed Tools Appl. 2024;83(24):64369–91. doi: 10.1007/s11042-024-18135-w

[10] Rahul, Katarya R. Deep auto encoder based on a transient search capsule network for student performance prediction. Multimed Tools Appl. 2022;82(15):23427–51. doi: 10.1007/s11042-022-14083-5

[11] Wang X, Zhao Y, Li C, Ren P. ProbSAP: a comprehensive and high-performance system for student academic performance prediction. Pattern Recognition. 2023;137:109309. doi: 10.1016/j.patcog.2023.109309

[12] Nakhipova V, Kerimbekov Y, Umarova Z, Bulbul H ibrahim, Suleimenova L, Adylbekova E. Integration of collaborative filtering into naive bayes method to enhance student performance prediction. International Journal of Information and Communication Technology Education. 2024;20(1):1–18. doi: 10.4018/ijicte.352512

[13] Yin W, Kann K, Yu M, Schutze H. Comparative study of CNN and RNN for natural language processing. arXiv preprint. 2017. p. 1–7. arXiv:1702.01923v1

[14] Márquez-Vera C, Cano A, Romero C, Noaman AYM, Mousa Fardoun H, Ventura S. Early dropout prediction using data mining: a case study with high school students. Expert Systems. 2015;33(1):107–24. doi: 10.1111/exsy.12135

[15] Thammasiri D, Delen D, Meesad P, Kasap N. A critical assessment of imbalanced class distribution problem: the case of predicting freshmen student attrition. Expert Systems with Applications. 2014;41(2):321–30. doi: 10.1016/j.eswa.2013.07.046

[16] Karimi H, Derr T, Huang J, Tang J. Online academic course performance prediction using relational graph convolutional neural network. In: Proceedings of the 13th International Conference on Educational Data Mining. 2020. p. 444–50.

[17] Kaura P, Singh M, Josan GS. Classification and prediction based data mining algorithms to predict slow learners in education sector. In: Proceedings of the 3rd International Conference on Recent Trends in Computing. 2015. p. 500–8.

[18] Aldowah H, Al-Samarraie H, Fauzy WM. Educational data mining and learning analytics for 21st century higher education: a review and synthesis. Telematics and Informatics. 2019;37:13–49. doi: 10.1016/j.tele.2019.01.007

[19] Ang KL-M, Ge FL, Seng KP. Big educational data & analytics: survey, architecture and challenges. IEEE Access. 2020;8:116392–414. doi: 10.1109/access.2020.2994561

[20] Hernández-Blanco A, Herrera-Flores B, Tomás D, Navarro-Colorado B. A systematic review of deep learning approaches to educational data mining. Complexity. 2019;2019(1):1–22. doi: 10.1155/2019/1306039

[21] Huang S, Fang N. Prediction of student academic performance in an engineering dynamics course: development and validation of multivariate regression models. International Journal of Engineering Education. 2010;26(4):1008–17.

[22] Huang S, Fang N. Predicting student academic performance in an engineering dynamics course: a comparison of four types of predictive mathematical models. Computers & Education. 2013;61:133–45. doi: 10.1016/j.compedu.2012.08.015

[23] Christian TM, Ayub M. Exploration of classification using NBTree for predicting students performance. In: Proceedings of the 2014 International Conference on Data and Software Engineering. Bandung, Indonesia: IEEE Computer Society; 2014. p. 1–6.

[24] Romero C, Espejo PG, Zafra A, Romero JR, Ventura S. Web usage mining for predicting final marks of students that use Moodle courses. Comp Applic In Engineering. 2013;21(1):135–46. doi: 10.1002/cae.20456

[25] Miguéis VL, Freitas A, Garcia PJV, Silva A. Early segmentation of students according to their academic performance: a predictive modelling approach. Decision Support Systems. 2018;115:36–51. doi: 10.1016/j.dss.2018.09.001

[26] Rizvi S, Rienties B, Khoja SA. The role of demographics in online learning; a decision tree based approach. Computers & Education. 2019;137:32–47. doi: 10.1016/j.compedu.2019.04.001

[27] Wasif M, Waheed H, Aljohani NR, Hassan SU. Understanding student learning behavior and predicting their performance. Cognitive Computing in Technology-Enhanced Learning. 2019;1(1):1–28.

[28] Young T, Hazarika D, Poria S, Cambria E. Recent trends in deep learning based natural language processing [review article]. IEEE Comput Intell Mag. 2018;13(3):55–75. doi: 10.1109/mci.2018.2840738

[29] Zhang L, Qi GJ, Wang L, Luo J. AET vs. AED: unsupervised representation learning by auto-encoding transformations rather than data. In: Proceedings of IEEE/CVF Conference on Computer Vision and Pattern Recognition. California, USA: IEEE/CVF; 2019. p. 2542–2550.

[30] Fazil M, Sah AK, Abulaish M. DeepSBD: a deep neural network model with attention mechanism for SocialBot detection. IEEE TransInformForensic Secur. 2021;16:4211–23. doi: 10.1109/tifs.2021.3102498

[31] Hassan S, Waheed H, Aljohani NR, Ali M, Ventura S, Herrera F. Virtual learning environment to predict withdrawal by leveraging deep learning. Int J Intell Syst. 2019;34(8):1935–52. doi: 10.1002/int.22129

[32] Botelho AF, Varatharaj A, Patikorn T, Doherty D, Adjei SA, Beck JE. Developing early detectors of student attrition and wheel spinning using deep learning. IEEE Trans Learning Technol. 2019;12(2):158–70. doi: 10.1109/tlt.2019.2912162

[33] Alam MM, Mohiuddin K, Das AK, Islam MdK, Kaonain MdS, Ali MdH. A Reduced feature based neural network approach to classify the category of students. In: Proceedings of the 2nd International Conference on Innovation in Artificial Intelligence. 2018. p. 28–32. 10.1145/3194206.3194218

[34] Raga RC, Raga JD. Early prediction of student performance in blended learning courses using deep neural networks. In: 2019 International Symposium on Educational Technology (ISET). 2019. p. 39–43. 10.1109/iset.2019.00018

[35] Hu Q, Rangwala H. Reliable deep grade prediction with uncertainty estimation. In: Proceedings of 9th International Conference on Learning Analytics and Knowledge. Arizona, USA: ACM; 2019. p. 76–85.

[36] Hai-tao P, Ming-qu F, Hong-bin Z, Bi-zhen Y, Jin-jiao L, Chun-fang L, et al. Predicting academic performance of students in Chinese-foreign cooperation in running schools with graph convolutional network. Neural Comput & Applic. 2020;33(2):637–45. doi: 10.1007/s00521-020-05045-9

[37] Junejo NUR, Huang Q, Dong X, Wang C, Humayoo M, Zheng G. SLPNet: student learning performance prediction during the COVID-19 pandemic via a deep neural network. In: Proceedings of CCF National Conference of Computer Applications, Harbin, China. 2024. p. 65–75.

[38] Junejo NUR, Huang Q, Dong X, Wang C, Zeb A, Humayoo M, et al. SAPPNet: students’ academic performance prediction during COVID-19 using neural network. Sci Rep. 2024;14(1):24605. doi: 10.1038/s41598-024-75242-2 39427025 PMC11490531

[39] Junejo NUR, Nawaz MW, Huang Q, Dong X, Wang C, Zheng G. Accurate multi-category student performance forecasting at early stages of online education using neural networks. Sci Rep. 2025;15(1):16251. doi: 10.1038/s41598-025-00256-3 40346097 PMC12064807

[40] Al-azazi FA, Ghurab M. ANN-LSTM: A deep learning model for early student performance prediction in MOOC. Heliyon. 2023;9(4):1–16. 10.1016/j.heliyon.2023.e15382 PMC10119763 37089306

[41] Hochreiter S, Schmidhuber J. Long short-term memory. Neural Computation. 1997;9(8):1735–80. 9377276 10.1162/neco.1997.9.8.1735

[42] Pascanu R, Gulcehre C, Cho K, Bengio Y. How to construct deep recurrent neural networks. In: Proceedings of the International Conference on Learning Representations. Banff, Canada: Arxiv; 2014. p. 1–13.

[43] LeCun Y, Bengio Y, Hinton G. Deep learning. Nature. 2015;521(7553):436–44. doi: 10.1038/nature14539 26017442

[44] Roy PK, Tripathy AK, Das TK, Gao X-Z. A framework for hate speech detection using deep convolutional neural network. IEEE Access. 2020;8:204951–62. doi: 10.1109/access.2020.3037073

[45] Yang Z, Yang D, Dyer C, He X, Smola A, Hovy E. Hierarchical attention networks for document classification. In: Proceedings of International Conference on 2016 Conference of the North American Chapter of the Association for Computational Linguistics: Human Language Technologies. California, USA: ACL; 2016. p. 1480–1489.

[46] Kuzilek J, Hlosta M, Zdrahal Z. Open university learning analytics dataset. Scientific Data. 2017;4(1):1–8. 10.1038/sdata.2017.171 PMC5704676 29182599

[47] Song X, Li J, Sun S, Yin H, Dawson P, Doss RRM. SEPN: a sequential engagement based academic performance prediction model. IEEE Intell Syst. 2021;36(1):46–53. doi: 10.1109/mis.2020.3006961

[48] Waheed H, Hassan S-U, Nawaz R, Aljohani NR, Chen G, Gasevic D. Early prediction of learners at risk in self-paced education: a neural network approach. Expert Systems with Applications. 2023;213:118868. doi: 10.1016/j.eswa.2022.118868

[49] Huang X, Khetan A, Cvitkovic M, Karnin Z. TabTransformer: tabular data modeling using contextual embeddings. arXiv prerpint 2020. p. 1–17. https://arxiv.org/abs/2012.06678

[50] Cholakov R, Kolev T. The GatedTabTransformer. An enhanced deep learning architecture for tabular modeling.

[51] Selwyn N. Re-imagining learning analytics a case for starting again? Internet and Higher Education. 2020;46(7):1–9.

