# Correction: MultiFAR: Multidimensional information fusion with attention-driven representation learning for student performance prediction

**DOI:** 10.1371/journal.pone.0340140

**Published:** 2026-01-02

**Authors:** Mohd Fazil, Bader M. Albahlal

The corresponding author’s email address (mohdfazil.jmi@gmail.com) should not have been included. The correct email address for Mohd Fazil is mfazil@imamu.edu.sa.
